# Comparative judgement as a research tool: A meta-analysis of application and reliability

**DOI:** 10.3758/s13428-025-02744-w

**Published:** 2025-07-10

**Authors:** George Kinnear, Ian Jones, Ben Davies

**Affiliations:** 1 School of Mathematics and Maxwell Institute for Mathematical Sciences, The University of Edinburgh, Edinburgh, UK; 2 Loughborough University, Loughborough, England; 3 University of Southampton, Southampton, UK

**Keywords:** Comparative judgement, Bradley–Terry, Reliability

## Abstract

Comparative judgement (CJ) provides methods for constructing measurement scales, by asking assessors to make a series of pairwise comparisons of the artefacts or representations to be scored. Researchers using CJ need to decide how many assessors to recruit and how many comparisons to collect. They also need to gauge the reliability of the resulting measurement scale, with two different estimates in widespread use: scale separation reliability (SSR) and split-halves reliability (SHR). Previous research has offered guidance on these issues, but with either limited empirical support or focused only on education research. In this paper, we offer guidance based on our analysis of 101 CJ datasets that we collated from previous research across a range of disciplines. We present two novel findings, with substantive implications for future CJ research. First, we find that collecting ten comparisons for every representation is generally sufficient; a more lenient guideline than previously published. Second, we conclude that SSR can serve as a reliable proxy for inter-rater reliability, but recommend that researchers use a higher threshold of .8, rather than the current standard of .7.

## Introduction

Applied psychological and behavioral research often depends on the construction of reliable measurement scales. A particular challenge arises in measuring participants’ preferences for various items or representations: asking participants to directly assign absolute scores (e.g., using a Likert scale) may prove unreliable because participants each interpret the absolute scores in different ways. Comparative judgement (CJ) is a method for constructing a measurement scale by asking participants to make relative judgements, in the form of paired comparisons (sometimes referred to as two-alternative forced choice or 2AFC). For example, Stadthagen-Gonzälez et al. (2019) used CJ to investigate English-Spanish bilinguals’ preferences for grammatical choices in different “code-switched” sentences, by presenting pairs of sentences and asking participants to “pick the one closer to the way they would speak to another bilingual person” (p. 210). After asking participants to make several of these paired comparisons, the data can be analysed using the Bradley–Terry model (Bradley & Terry, 1952) to generate scores for each of the artefacts or representations being compared. Thus, CJ provides a method for researchers to measure the qualities of a set of representations, which is important across a range of application areas (Issa Mattos & Martins Silva Ramos, 2022).

Researchers have noted that CJ can play an especially important role in measuring nebulous, hard-to-specify constructs. This has been a key strength in education research (Jones & Davies, 2024), where CJ has facilitated the measurement of constructs like conceptual understanding (Bisson et al., 2016) and the quality of instructional explanations (Evans et al., 2022). It has also proved useful in other fields; for instance, Zucco et al. (2019) used CJ to measure the relative worth of cabinet positions in the Brazilian government, by presenting 142 legislators with different pairs of ministries and asking them to select in each case “the position you think your party would prefer” in a coalition government. The researchers noted that the CJ results provided “information beyond what objective indicators can reveal” (Zucco et al., 2019, p. 6).

Despite widespread use of CJ methods, little is known about the range of approaches used by different researchers. The design of a CJ study entails many choices, including the number of representations to be compared, how many assessors (or judges) to recruit, and the number of comparisons each assessor should complete. Verhavert et al. (2019) analysed 49 education studies and included details of these numbers; for instance, they observed that the studies typically either involved judgement of many representations by a few expert assessors, or peer assessment where the number of representations and assessors was similar. A similar picture emerged from Bartholomew and Jones ’s (2022) systematic review that identified 21 studies of CJ as an assessment tool in higher education. They focused on studies using an adaptive algorithm for selecting the CJ pairings (known as ACJ, and discussed in more detail in the next section). These two studies provide insight into the way that education researchers have been using the CJ method; however, we are aware of no prior attempt to systematically review the full range of published studies using the CJ method.

Moreover, there is debate over how to evaluate the reliability of comparative judgement outcomes. One of the main ways of measuring reliability in CJ research is using the scale separation reliability (SSR). This statistic is generated along with the scores for the representations when fitting the Bradley–Terry model, and is conceptually related to Cronbach’s alpha (Bramley, 2007). However, the proper interpretation of the SSR measure is not entirely clear (Verhavert et al., 2018), and other researchers have advocated for the more transparent split-halves reliability (SHR) measure, which is consistent with the approach taken in other applied psychological research (Jones & Davies, 2024). Two recent meta-analyses have investigated SSR, including the relationship between SSR and SHR. Verhavert et al. (2018) examined data from 26 CJ studies, and concluded that SSR was a good estimate of the split-halves reliability. Accordingly, the meta-analysis of 49 CJ studies by Verhavert et al. (2019) focused only on SSR, and investigated the relationship between characteristics of the study design and SSR. They found that the number of comparisons per representation was a significant predictor of SSR, and identified threshold values to guide researchers designing CJ studies. However, the work to date has been focused on applications in education, and with relatively small samples, so it remains to be seen if the findings translate to more diverse study designs.

In this paper, we describe the ranges of assessors, representations and comparisons used in comparative judgement studies and the reliability of reported outcomes. We also take the opportunity to re-analyse the data collected from across multiple studies to investigate how different measures of the reliability of outcomes relate to one another. Our findings help to establish guidelines for researchers about the design of CJ studies.

## Comparative judgement

Comparative judgement (CJ) is a general method for constructing measurement scales that is often associated with Thurstone (1927). CJ involves three key components, which we refer to using the same terms as Verhavert et al. (2018). First are the *representations*; these are the artefacts we wish to position on a scale, which can be anything from samples of students’ writing (e.g., Wheadon et al., 2020), through to samples of cold-brew coffee (e.g., Luckett et al., 2020), or even images taken by spacecraft (Jones et al., 2020). Second are the *comparisons*; these are the pairings of the representations, each of which requires a binary decision as to which is ‘better’ or the ‘winner’. Finally, there are the judges or *assessors*; these are the people who make the comparisons.

The main outcome of a CJ study is a set of comparisons. Each row contains information about which two representations were paired, which representation ‘won’, and which assessor made the comparison. The set of comparisons is then processed to produce a score for each representation. There are different possible methods; for example, we could simply count each representation’s number of wins and use these counts as scores. However, the standard method is to fit a model to the comparison data, and the most commonly used is the Bradley–Terry model (Hamilton et al., 2023).

The technicalities of fitting the Bradley–Terry model have been discussed extensively elsewhere (e.g., Bramley, 2007; Jones & Davies, 2024; Pollitt, 2012). Here, we highlight three key features of the outcomes as implemented by the R package that we use, sirt (Robitzsch, 2024)^1^. First, each representation is assigned a unique relative score and this differs from common methods such as using Likert scales where several representations may get the same score. Second, the modelling produces a set of scores that have a mean of approximately $0$, with the standard deviation typically ranging from around $1.5$ to $3.5$ (Bramley & Vitello, 2018). Third, each score comes with a standard error, which gives a measure of the precision in the estimate of that score.

In the 20th century, comparative judgement studies often collected a decision for every possible pairing of representations. However, this limited researchers to constructing scales of only small numbers of representations because the number of possible pairings increases proportionally to the square of the total representations. Many contemporary studies do not use all possible pairings, and therefore an algorithm is required to select which pairings to present to assessors. There are two contrasting approaches (Jones & Davies, 2024). One is to pair representations randomly. In practice, the pairing is often pseudo-random, according to constraints such as ensuring representations all receive about the same number of comparisons, that each representation is viewed by different assessors, and so on. The other approach is to use an adaptive algorithm, where selection is informed by the comparisons already completed (Pollitt, 2012). Advocates for adaptive algorithms argue that they reduce the number of comparisons needed to construct a reliable measurement scale (e.g., Pollitt, 2012). However, critics point to evidence that adaptive algorithms inflate reliability (e.g., Bramley & Vitello, 2018), as we discuss later.

### Estimating reliability

An important step in constructing a measurement scale is estimating its reliability; this is, the extent to which the variability in scores is due to genuine differences rather than measurement error (Wright & Masters, 1982). Common social science methods for estimating reliability include Cronbach’s $\alpha$ and inter-rater reliability. When constructing a scale using CJ methods, the commonly used estimates are scale separation reliability and split-halves reliability. We introduce these in the next two sections, before moving on to an overview of research into their features.

#### Scale separation reliability (SSR)

SSR is the most widely reported reliability estimate for outcomes of the Bradley–Terry model, and is derived from expected and observed scores. The expected scores – known as the ‘true’ scores in educational assessment literature – are assumed to be precise and stable but unknowable. The observed scores are assumed to deviate from the true scores by a random error (Bond & Fox, 2007). In practice, the observed scores are obtained by fitting the Bradley–Terry model to the decision data, and the true scores are obtained by subtracting the error term from the observed scores (Bramley & Vitello, 2018). The error term is the mean of the squares of the standard errors of the scores, often denoted $MSE$. SSR is calculated as the proportion of variance in the observed scores accounted for by the variance of the true scores,

1 $$\begin{aligned} SSR = \dfrac{SD^2 - MSE}{SD^2} \end{aligned}$$

where $SD^2$ is the variance of the observed scores. SSR statistics vary from $0$ to $1$ with values $>.7$ considered acceptable and values $>.8$ or $>.9$ considered high or very high (Verhavert et al., 2018).

A drawback of SSR is a lack of clarity and consistency in how it is conceived by different researchers. It is commonly claimed to be analogous to Cronbach’s $\alpha$ and therefore conceived as a measure of internal consistency (e.g., Bramley, 2007). It has also been considered a proxy for inter-rater reliability (e.g., Verhavert et al., 2019), analogous to two raters independently scoring the representations and correlating their scores. This lack of clarity has driven some researchers to also report a newer estimate of the reliability of CJ outcomes, often referred to as split-halves reliability (Bisson et al., 2016).

#### Split-halves reliability (SHR)

SHR was introduced to provide an estimate of the reliability of scales constructed using CJ that is comparable to inter-rater reliability. It is based on correlating the observed scores obtained from independent groups of assessors making comparisons of the same set of representations. For example, in Jones et al. (2014) two independent groups of education experts comparatively judged the same mathematics exam scripts. The Bradley–Terry model was fitted to each set of comparisons, thereby producing two independent sets of scores for the scripts. The authors reported what they called ‘inter-rater reliability’ (p. 161), which was based on computing the Pearson product–-moment correlation coefficient between the two sets of scores.

More commonly, authors use a post hoc inter-rater reliability introduced by Jones et al. (2013), often called split-halves reliability. SHR is calculated once all assessors have completed their comparisons. The assessors are then randomly split into two subgroups, and the Bradley–Terry model is fitted to the comparisons of each subgroup. The Pearson correlation coefficient is then calculated using the two independent sets of scores. This random splitting procedure is repeated (typically $100$ or $1000$ times), to produce a set of correlation coefficients. The mean or median correlation coefficient is then taken as an estimate of the inter-rater reliability of the CJ scores.

Reporting SHR is attractive because of its conceptual familiarity to social scientists, in contrast to the lack of clarity and consistency associated with SSR. Indeed, a similar post hoc approach to inter-rater reliability is commonly used in social science (e.g., Zhang et al., 2024). However, a disadvantage of SHR is that it underestimates reliability. This is because splitting the assessors into two subgroups results in scores based on only half the available data (Bisson et al., 2016). One way to address the conservatism of the SHR measure is by doubling the number of assessors. For instance, Jones et al. (2019, p. 674) recommended “ten judgements per response to ensure a reasonable SSR, and therefore 20 judgements per response are required to evaluate inter-rater reliability”. However, this dramatically increases the resources required to use CJ. In practice, recruiting assessors can be difficult, time-consuming and expensive, so doubling the number of assessors may not be feasible.

### Reliability research

Recently, researchers interested in CJ methods have investigated the accuracy of different estimates of reliability, as well as the study design decisions that can influence the reliability of outcomes. For instance, Bramley and Vitello (2018) demonstrated that SSR estimates are artificially inflated when using an adaptive algorithm to pair representations; this finding has led many researchers to avoid using adaptive algorithms.

#### Relationship between SSR and SHR

The relationship between the SSR and SHR measures has been of particular interest. Verhavert et al. (2018) compared SSR and SHR for 26 sets of non-adaptive CJ decision data and concluded that “SSR is a quite good estimate of the split-half correlation” (p. 435). They argued that further research is needed to establish whether SSR can act as a proxy for SHR, thereby saving the time and resources needed to estimate the latter. This is one of the motivations for the present study.

#### Factors affecting reliability

Verhavert et al. (2019) looked at six characteristics of CJ studies that potentially influence SSR in a sample of 49 sets of comparison data. Three of these characteristics are relevant here^2^, along with two measures derived from them:

- $N_R$, the total number of representations to be assessed, which ranged from 6 to 1089 ($M=84$).
- $N_C$, the total number of comparisons, which ranged from 54 to 9038 ($M=817$).
- $N_A$, the total number of assessors, which ranged from 4 to 127 ($M=29$).
- $N_{RA}=\dfrac{N_R}{N_A}$, the number of representations per assessor.
- $N_{CR}=2\dfrac{N_C}{N_R}$, the number of comparisons per representation, where the factor of $2$ reflects the fact that each comparison features two representations. By including the factor of 2, $N_{CR}$ gives the average number of comparisons involving any particular representation. Alternatively, for the purposes of designing a research study, it can be helpful to think about a multiplier for the number of comparisons to collect for each representation, $CPR=\frac{N_C}{N_R}=\frac{1}{2} N_{CR}$.

Verhavert et al. (2019) found that among these characteristics, only $N_{CR}$ had a significant relationship with SSR. Based on these findings, Verhavert et al. (2019) offered a tentative guideline to researchers seeking to construct a measurement scale using CJ: “between 10 and 14 comparisons per representation are needed to reach a reliability of .70. To reach a reliability of .90, 26 to 37 comparisons per representation are needed.” (p. 557).

Previously, researchers had offered recommendations about $CPR$, based on their experience and intuitions (summarised in Table 1). For instance, Wheadon (2015) recommended “multiplying the number of scripts you have by 5” (i.e., $CPR\ge 5$ or $N_{CR}\ge 10$), and Bisson et al. (2016, p. 154) suggested “at least ten times the number of judgements to the number of scripts” (i.e., $CPR\ge 10$ or $N_{CR}\ge 20$). Verhavert et al.’s (2019) empirically grounded recommendations correspond to $CPR\ge 5$ to reach a reliability of .7 and $CPR\ge 13$ to reach a reliability of .9. One aim of our study is to validate this advice using a larger sample of CJ studies.

**Table 1:** Summary of previous recommended thresholds for the number of comparisons per representation

Source	Recommendation for $N_{CR}$
Wheadon (2015)	$N_{CR}\ge 10$
Bisson et al. (2016)	$N_{CR}\ge 20$
Verhavert et al. (2019)	$10\le N_{CR}\le 14$ for SSR of .70
	$26\le N_{CR}\le 37$ for SSR of .90
Crompvoets et al. (2021)	$N_{CR}\ge 41$

Rather than considering existing empirical CJ datasets, Crompvoets et al. (2021) used simulation methods to benchmark SSR against true reliability (which is of course unknown for empirical datasets). Their results suggested that, for large $N_{CR}$, SSR was an accurate estimate of true reliability across a range of $N_R$ between 20 and 100, and score variances between 0 and 3. They also found that $N_{CR}$ could be reduced without loss of SSR accuracy, especially for larger $N_R$, and larger true score variance. Based on the simulation evidence, they recommended $N_{CR}\ge 41$ to obtain an accurate SSR estimate. Interestingly, this recommended threshold for an accurate SSR is similar to Verhavert et al. (2019)’s recommended $N_{CR}\ge 37$ to obtain SSR $\ge .90$.

#### Correcting SHR

Regarding SHR, researchers’ intuition that the split-halves method underestimates reliability (e.g., Bisson et al., 2016; Jones et al., 2019) has been confirmed using simulation methods: Hamilton (2023, p. 124) found that “the agreement we might expect ... is substantially higher than the split-half method suggests.” The conservatism of the split-halves method has been noted in other (non-CJ) contexts where it is used to estimate reliability; for instance, Williams and Kaufmann (2012, p. 889) noted that “reliability coefficients from split-half estimates ... underestimate the reliability” of an association task. They found that “a good empirical approximation” (Williams & Kaufmann, 2012, p. 889) of the correct reliability value was provided by the Spearman–Brown prophecy formula – despite the fact that the rationale for that formula only applies to the context of tests made up of items, as a way to “extrapolate the test–retest reliability from the split-half reliability” (Luna et al., 2021, p. 1130). For an SHR estimate of $\rho$, the Spearman–Brown correction suggests the test-retest reliability is $\frac{2\rho }{1+\rho }$. It remains to be seen whether the Spearman–Brown formula can correct the underestimate in SHR, and we return to this question in our analysis.

## Research focus

The present meta-analysis contributes to contemporary research on the interpretation of the SSR and SHR reliability measures, and their relationship with study characteristics. Previous meta-analyses only considered data from education studies carried out on one particular CJ platform (Verhavert et al., 2018, 2019). In contrast, in the present study our meta-analysis includes data from a wide range of disciplines and research groups, with CJ studies that used various types of representations and assessors. The dataset enabled us to map the variation of published CJ studies in terms of characteristics including $N_R$, $N_C$ and $N_A$, as well as reliability statistics, and to explore the relationships between characteristics and reliability.

Our research questions are:

1. How do study characteristics (number of assessors, representations and comparisons) and reliability measures (SSR and SHR) vary across CJ studies?
2. How do study characteristics influence reliability?
3. How does SSR function as a measure of inter-rater reliability?

**Fig. 1:**
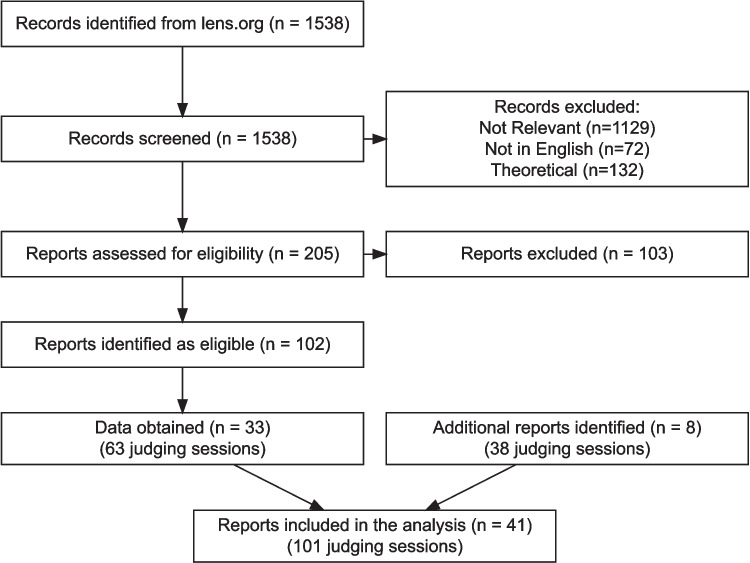
Flowchart summarising the data collection process

## Method

To address our research questions, we conducted a meta-analysis of studies that used CJ. We restricted our analysis to studies where we could obtain the original decision data, so that we could fit the Bradley–Terry model and calculate both SSR and SHR for each study. This was necessary since studies do not always report these values (particularly SHR), and since reported values of SSR may have been based on an incorrect formula.

### Literature search

Our process for identifying studies to include in the meta-analysis is graphically summarised in Fig. 1. We used the lens.org database (which aggregates the CrossRef, PubMed, OpenAlex and Microsoft Academic databases) to identify published work that may employ CJ, with the query:

("comparative judgement" OR "comparative judgment")

OR

(("pairwise comparisons" OR "paired comparisons") AND "Bradley" AND "Terry").

These search terms were chosen to reflect known differences in the way CJ research is reported across different fields. We filtered the search results to those published in 2010 or later (the search was run on 7th June 2021), resulting in a total of 1538 papers.

### Screening

Our inclusion criteria required that the article reported on comparative judgement data generated by human judges. We screened our search results using three preliminary exclusion criteria. First, we excluded 72 papers that were not published in English. We acknowledge this as a non-trivial limitation of our work, which may bias our findings, but was necessary given the expertise and resources of the research team. Second, we excluded 132 papers that we deemed to be ‘theoretical’ in nature. These were papers primarily focused on the statistical methodology of generating scores from binary decisions, with non-novel datasets being used mainly to illustrate methods (e.g., the preprint of Mattos & Silva Ramos, 2022, was excluded for this reason). Third, we excluded 1129 papers that we deemed not relevant. Many were excluded because they used the phrase ‘comparative judgement’ but not in reference to the paired comparison method. Others were excluded because they were not empirical CJ studies with human assessors, or because they did not process the decision data to produce scores for the representations.

The third author screened all 1538 papers according to these exclusion criteria (primarily using title and abstract, but consulting the full text where necessary). To evaluate the reliability of the screening process, the other two authors independently screened 10% (5% each) of the excluded papers and the decisions were confirmed in all but five cases. For those five papers, a closer inspection and discussion resulted in their inclusion. This left 205 papers which were divided between the authors for the final screening process, in which the full text of each paper was read to determine whether it was within scope. This resulted in a further 103 exclusions, with 102 papers remaining within our scope.

### Requesting data

For each of the 102 papers that we identified as eligible, we e-mailed the corresponding author in August 2021 to request the data (see https://osf.io/6gmvh for the email text). We sent a follow-up email to those who did not reply within 8 weeks, advising that we would cease data collection within 3–4 weeks.

By the end of the process, we obtained data from 33 of the 102 eligible publications. We received replies from the authors of 44 of the remaining publications, indicating they were unable to provide the requested data, with most of those explaining that the data were no longer available^3^.

Our email to authors also included a request for other relevant data that had either not been detected by our search, or had never been published. This snowballing approach added a further eight reports to our analysis.

Thus, our analysis is based on data from $n = 41$ papers for which we could obtain the data. Since some of the papers reported on multiple CJ sessions, we obtained data from a total of 101 judgement sessions.

**Table 2:** Summary of the features of the comparative judgement sessions in our sample

Characteristic	Overall, *N* = 101	Search, *N* = 63	Snowballing, *N* = 38
Adaptive CJ			
No	64 (63%)	41 (65%)	23 (61%)
Yes	19 (19%)	19 (30%)	0 (0%)
Unknown	18 (18%)	3 (4.8%)	15 (39%)
Research topic			
Education	77 (76%)	44 (70%)	33 (87%)
Other	24 (24%)	19 (30%)	5 (13%)
Open data	25 (25%)	18 (29%)	7 (18%)

EMPTY

### Data cleaning and preparation

For each of the 101 judgement sessions, we assembled the CJ decision data in a standard format in the openly available repository at https://osf.io/m5wtz/. We also recorded details of each judgement session (including whether adaptive CJ was used) based on our reading of the published report. A summary of these details is shown in Table 2. In particular, while the previous meta-analysis by Verhavert et al. (2019) was based entirely on CJ studies in an education context, our sample includes 24 datasets from other research fields. Nevertheless, our sample is dominated by education studies; we return to this point in the Discussion. We identified that a majority (63%) of judgement sessions used non-adaptive CJ, while for 18% of judgement sessions we could not determine whether adaptivity was used. Only a minority of datasets (25%) were already openly available.

For each judgement session, we computed the characteristics ($N_A$, $N_C$ and $N_R$) from the raw CJ decision data. We also computed the SSR and SHR reliability measures, rather than relying on reported values since those were not always available. Moreover, this ensured that the reliability values were computed accurately and consistently across all judgement sessions, as follows:

1. scale separation reliability (SSR), given by the formula^4^$SSR=\frac{G^2}{1+G^2}$, where $G$ is the value sepG computed by sirt::btm (Robitzsch, 2024),
2. Split-halves reliability (SHR), where the assessors are randomly split into two groups and the resulting scores from the two groups are correlated; we report the median Pearson correlation from 100 iterations of this procedure.

### Analysis

We defer a detailed description of our analysis methods to the point where they arise in the Results. In brief, we address each question as follows:

- RQ1: How do study characteristics and reliability measures vary across CJ studies? We visualise the distribution of the various measures, and use descriptive statistics such as the range and median to describe key features.
- RQ2: How do study characteristics influence reliability? For each of SSR and SHR, we carry out a regression analysis with study characteristics as predictors of the reliability measure.
- RQ3: How does SSR function as a measure of inter-rater reliability? Using SHR as a proxy for inter-rater reliability, we use correlation and regression to compare SSR to SHR, and to Spearman–Brown-corrected SHR. Finally, we simulate a comparison between SSR and true inter-rater reliability, by splitting the assessors in each judgement session into two groups at random and comparing the SSR for one (split-half) group with the inter-group correlation (and taking the median of each measure over 100 iterations).

## Results

### RQ1: How do study characteristics and reliability measures vary across CJ studies?

#### Study characteristics

**Fig. 2:**
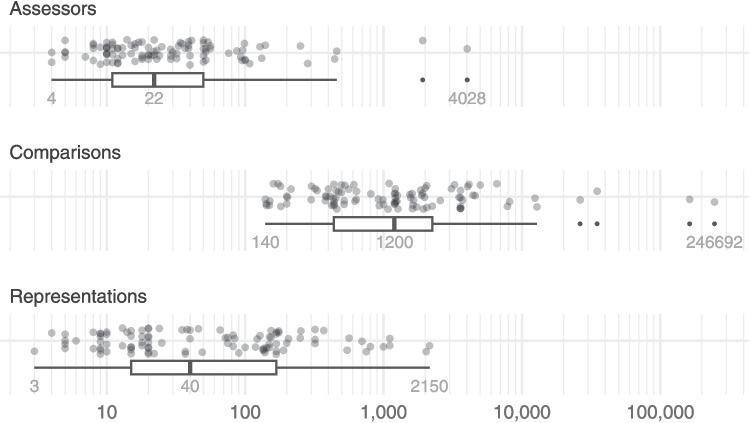
$N_A$, $N_C$ and $N_R$ across the judgement sessions, summarised by boxplots showing the minimum, median and maximum values. Note that these are shown on a logarithmic scale, and outliers in the boxplots are shown as points

The numbers of representations, assessors and comparisons ranged widely across the judgement sessions, as shown in Fig. 2. The range of the number of representations was 2147. Only four judgement sessions (4%) had more than 1000 representations and most (60%) had fewer than 100 representations (the median was 40). The range of the number of assessors was 4024, although most judgement sessions (84%) had fewer than 100 assessors (the median was 22). The two judgement sessions with over 1000 assessors were examples of “citizen science”, where members of the public were asked to compare images of coronal mass ejections (Jones et al., 2020). In contrast, among the 21 judgement sessions with no more than ten assessors, most (76%) used expert assessors; the others were gathering layperson/novice judgements to compare them to expert judgements. The number of comparisons ranged from 140 to 246,552. All judgement sessions had more than 100 comparisons and only six (6%) had more than 10,000 comparisons.

**Fig. 3:**
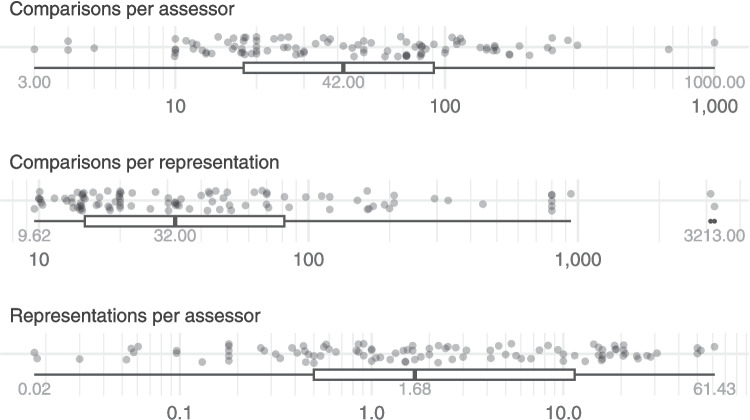
Ratios of the quantities from Fig. 2 for each judgement session, summarised by boxplots showing the minimum, median and maximum values. Note that these are shown on a logarithmic scale, and outliers in the boxplots are shown as points

#### Relationships between study characteristics

To investigate relationships between the three study characteristics, we considered the distribution of their ratios (in line with Verhavert et al., 2019). Figure 3 shows the numbers of representations per assessor, comparisons per assessor and comparisons per representation^5^ within each judgement session on a logarithmic scale. The range of the number of representations per assessor was 61.41 although the median was only 1.68 and most judgement sessions (74%) had fewer than ten representations per assessor. This reflects the fact that many of the judgement sessions had a large group of assessors comparing a relatively small set of items; for instance, the two judgement sessions from Mejía Ramos et al. (2021) involved two different groups of mathematicians (22 from one university and 16 from another) making comparisons of the same set of nine mathematical proofs (giving $N_{RA}$ of $.41$ and $.56$ respectively). The number of comparisons per assessor varied over a large range; from 3 (Luckett et al. (2020), where each assessor taste-tested three pairs of cold-brew coffee drinks) to 1000 (Ofqual (2015), where PhD students were employed to compare the difficulty of mathematics exam questions, and each completed 1000 comparisons). Most judgement sessions (76%) had fewer than 100 comparisons per assessor, with a median of 42. While some judgement sessions were implemented with each assessor making the same, fixed number of comparisons (as in Luckett et al., 2020; Ofqual, 2015), other judgement sessions allowed assessors to choose how many comparisons to make (e.g., Hunter and Jones (2018, p. 3) reported school teachers making “between 72 and 1400 judgements each”). The number of comparisons per representation ranged from 9.62 to 3213, with only one judgement session narrowly below the minimum of 10 comparisons per representation recommended by Wheadon (2015). Many of the judgement sessions surpassed other recommended thresholds, with 63% having $N_{CR}\ge 20$ and 44% having $N_{CR}\ge 37$.

**Fig. 4:**
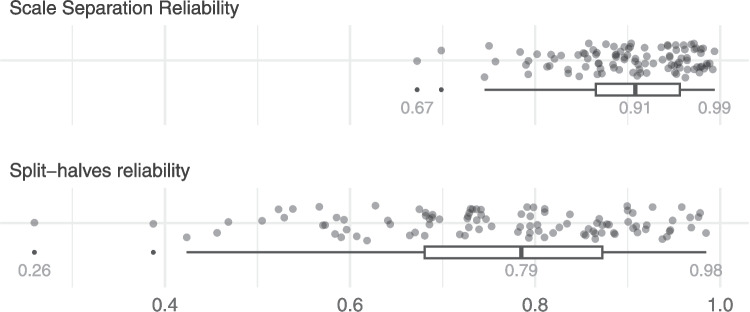
The observed distribution of reliability measures across all the judgement sessions, summarised by boxplots

#### Reliability measures

For each judgement session, we computed the SSR and SHR measures using the original comparison data. These values are shown in Fig. 4 for all the judgement sessions. The SSR values ranged from .51 to .99, while SHR ranged from .26 to .99. The SSR values tended to be higher than SHR, with a median value of .91 for SSR, compared with .79 for SHR. Most SSR values (95%) were above the commonly used threshold of .70, while only 67% of the SHR values were above .70.

### RQ2: How do study characteristics influence reliability?

#### Investigating the influence of adaptivity

**Fig. 5:**
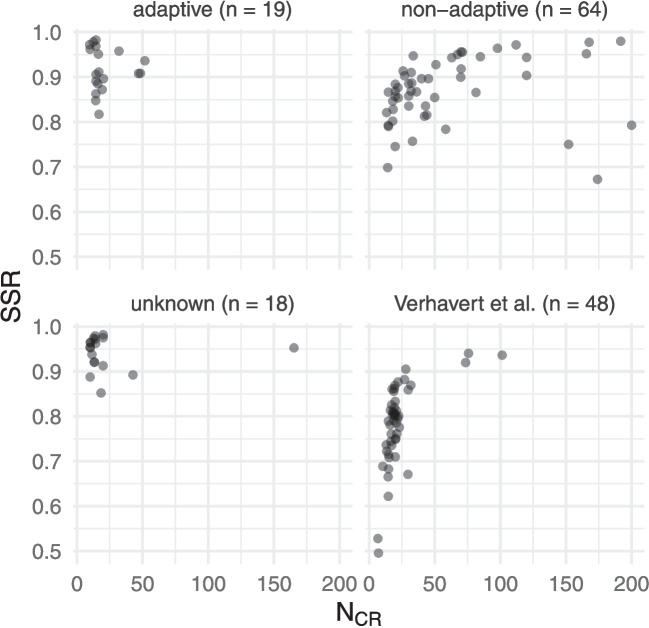
The number of comparisons per representation compared with the SSR for each judgement session, with an indication of whether the judgement session used an adaptive CJ algorithm. Values from the (non-adaptive) CJ analysed by Verhavert et al. (2019) are also shown for reference. Note that 14 non-adaptive judgement sessions with $N_{CR}\ge 200$ have been omitted for clarity; all of these had $SSR\ge .87$

Previous research established that SSR was inflated by adaptivity (Bramley & Vitello, 2018). The previous meta-analysis by Verhavert et al. (2019) only included data from non-adaptive CJ sessions, so did not need to account for adaptivity as a possible factor influencing SSR. Our sample included data from a mix of adaptive and non-adaptive CJ sessions, so we first sought to check whether adaptivity influenced SSR. Figure 5 shows the SSR values for each judgement session, compared with the $N_{CR}$ values (expanding on Figure 4 of Verhavert et al., 2019). The adaptive and non-adaptive judgement sessions form clear clusters, with adaptive judgement sessions evidencing inflated SSR values for similar $N_{CR}$ levels. (As shown in Fig. 5, the 19 adaptive judgement sessions had $N_{CR}$ below 52 and had mean SSR of .92; for the 32 non-adaptive judgement sessions with $N_{CR}$ below 52, the mean SSR was .85). We therefore focus on the subset of 64 non-adaptive CJ sessions in the remainder of this section.

**Table 3:** Results of the linear regressions with SSR and SHR as the response variables

	Model fit		Coefficients				
Model	$R^2$	$p$	Term	Estimate	SE	$t$	$p$
SSR	0.37	$<0.001$	Intercept	0.69	0.04	18.92	$<0.001$
			$\log (N_A)$	-0.02	0.02	-0.75	.457
			$\log (N_C)$	0.10	0.02	4.03	$<0.001$
			$\log (N_R)$	-0.06	0.02	-3.11	.003
SHR	0.32	$<0.001$	Intercept	0.43	0.09	4.94	$<0.001$
			$\log (N_A)$	0.08	0.06	1.42	.161
			$\log (N_C)$	0.11	0.06	1.82	.074
			$\log (N_R)$	-0.09	0.04	-2.05	.044
SSR	0.37	$<0.001$	Intercept	0.70	0.04	19.70	$<0.001$
			$\log (N_C)$	0.08	0.01	5.91	$<0.001$
			$\log (N_R)$	-0.05	0.01	-3.33	.001
SHR	0.30	$<0.001$	Intercept	0.40	0.09	4.70	$<0.001$
			$\log (N_C)$	0.17	0.03	5.08	$<0.001$
			$\log (N_R)$	-0.12	0.03	-3.56	$<0.001$
SSR	0.28	$<0.001$	Intercept	0.76	0.03	28.28	$<0.001$
			$\log (N_{CR})$	0.07	0.01	4.94	$<0.001$
SHR	0.27	$<0.001$	Intercept	0.46	0.06	7.52	$<0.001$
			$\log (N_{CR})$	0.15	0.03	4.78	$<0.001$

Note that base 10 logarithms have been applied to the predictors, and the coefficients are unstandardised

#### Identifying characteristics that influence reliability

To explore the influence of the study characteristics on reliability, we used separate linear regression models with the study characteristics as predictors and the reliability measures (SSR and SHR) as the response variables. Since our earlier exploration of the study characteristics showed that they are best presented on a logarithmic scale (as in Fig. 2), we applied (base 10) logarithms to these predictors in the regression model. To avoid introducing collinearity in the predictors,^6^ we focused on the three characteristics $N_{A}$, $N_C$ and $N_R$. We used a stepwise procedure starting with the full model, and reducing it as necessary (by removing the least significant predictor) to arrive at the simplest possible model. The full results are shown in Table 3.

**Fig. 6:**
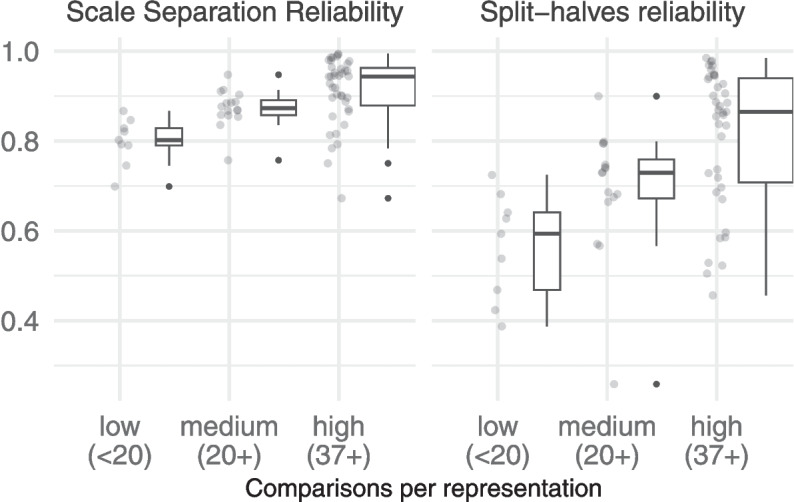
The observed distribution of reliability measures across the non-adaptive judgement sessions, summarised by boxplots, for different levels of comparisons per representation

The regression models with all three predictors provided a reasonably good fit, with the explained variance ($R^2$) being 37% for SSR and 32% for SHR. However, not all of the predictors were significant in these models; in each case, $\log (N_A)$ had the highest $p$-value, so we removed it. The regression models with only $\log (N_C)$ and $\log (N_R)$ as predictors also provided a reasonably good fit, with the explained variance only very slightly reduced from those of the full model (37% for SSR and 30% for SHR). In both models, the two predictors were significant, so these are the models that we interpret.

For each of SSR and SHR, the coefficient of $\log (N_C)$ was positive and $\log (N_R)$ was negative; that is, a higher number of comparisons is associated with increased reliability, while a higher number of representations is associated with decreased reliability (assuming all other characteristics are held constant). The interpretation of the coefficients takes some care due to the log transformations. For instance, for SSR the coefficient of $\log ({N_C})$ is 0.08; thus, if all other characteristics are held constant, the model predicts that increasing the number of comparisons by 10% (i.e., multiplying by 1.1) would increase the SSR by $0.08 \times \log _{10}(1.1)=0.003$.

In previous research, Verhavert et al. (2019) identified $N_{CR}$ as the single best predictor of reliability. Note that using $\log (N_{CR})$ as a predictor is equivalent to having both $\log (N_C)$ and $\log (N_R)$ as predictors (as in our final model), but with an added constraint on their coefficients: since $\log (N_{CR})=\log 2+\log (N_C)-\log (N_R)$, the coefficients of the predictors are constrained to be equal in magnitude but differing in sign. In our results, the coefficients are not exactly equal in magnitude; however, they are reasonably close, so we investigated how well the model would perform using only $\log (N_{CR})$ as a predictor. The model with $\log (N_{CR})$ as the only predictor explained a substantial proportion of the variance: 28% for SSR and 27% for SHR. Thus, among the study characteristics, $N_{CR}$ does seem to play an important role in influencing reliability.

#### Comparisons per representation

Given that $N_{CR}$ predicts both SSR and SHR, and has been the basis for existing guidance for the design of CJ studies, we also investigated how reliability varied across the 64 non-adaptive judgement sessions, grouped by the recommended thresholds of $N_{CR}\ge 20$ (Bisson et al., 2016) and $N_{CR}\ge 37$ (Verhavert et al., 2019). We created three groups with low, medium and high $N_{CR}$, as shown in Fig. 6. A Kruskal–Wallis test on the SSR values of the three groups showed that the differences were significant, $H(2,64)=19.23, p<.001$. Post hoc comparisons using Dunn’s test with Holm–Bonferroni correction revealed no significant difference between the low and medium groups ($z=2.13, p=.051$), or between the medium and high groups ($z=2.23, p=.051$). Similarly, a Kruskal–Wallis test of the SHR values across the three groups was significant, $H(2,64)=17.67, p<.001$. Again, post hoc comparisons revealed no significant difference between the low and medium groups ($z=1.73, p=.084$), but there was a significant difference between the medium and high groups ($z=2.43, p=.030$).

There are three outliers visible in Fig. 5, with SSR below .8 despite having $N_{CR}$ over 100. All three of these judgement sessions come from Luckett et al. (2020), where laypeople compared samples of cold-brew coffee, ice cream, and pizza. Luckett et al. (2020, p. 3) noted that it is “well known that consumer liking for food products is not always in high agreement”, which may explain these low SSR values. (A fourth study involving cola drinks had higher SSR; in that study, assessors made ten comparisons rather than only three for the coffee or five for the ice cream and pizza.)

### RQ3: How does SSR function as a measure of inter-rater reliability?

We considered two ways to evaluate SSR as a proxy measure for inter-rater reliability. First, we compared the values of SSR and SHR across the judgement sessions in our sample, since SHR is well-understood as a measure of inter-rater reliability. In addition, since SHR in effect gives an estimate of the inter-rater reliability of a group of assessors half the size of the actual group, we also compared SSR with the Spearman–Brown-corrected version of SHR. The second approach to evaluating SSR was to carry out a simulation study based on the datasets. As in the previous section, the analyses here were focused on the 64 non-adaptive CJ sessions.

**Fig. 7:**
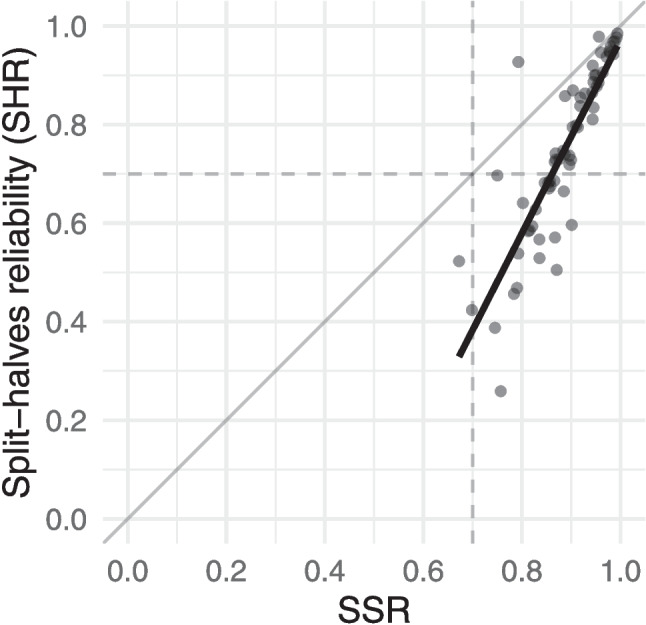
Values of SSR compared with SHR for the 64 non-adaptive judgement sessions in our sample. The plot includes the regression (*thick line*) as well as the identity (*thin line*) and the commonly used thresholds of .7 (*dashed line*)

**Fig. 8:**
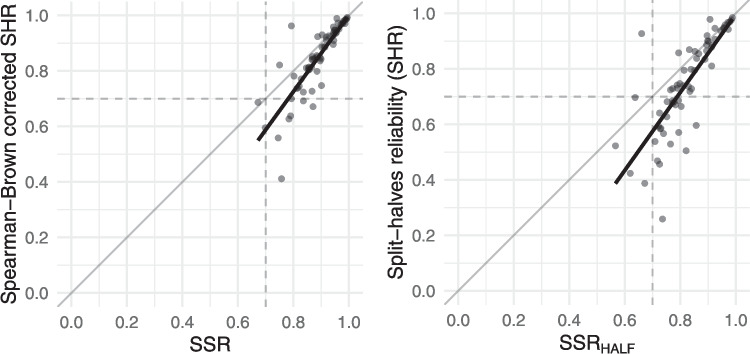
Two alternative ways of viewing the SSR–SHR relationship. **a** Comparing SSR with Spearman–Brown-corrected SHR. **b** Comparing $\text {SSR}_\text {HALF}$ with SHR. Each plot includes the regression (thick line) as well as the identity (*thin line*) and the commonly used threshold of .7 (*dashed line*)

#### Comparing SSR with SHR

For the 64 non-adaptive judgement sessions in our sample, SSR and SHR were highly correlated, $r=.86$ (95% CI $[.78, .91]$). The linear regression shown in Fig. 7 was also significant ($p<.001$, with $R^2=.74$) and gave the best-fitting line as $\text {SHR} = 1.961\times \text {SSR}-0.990$.

Despite evidence of a strong relationship, Figure 7 also shows there were outliers. For instance, the judgement sessions with the lowest observed SHR of .26 had an SSR of .76. This was from an unpublished study and used only nine novice assessors, so the SHR could have been hampered by low number of judgements as it is particularly sensitive to any differences of opinions between the judges. Indeed, Bisson et al. (2016, p. 154) suggested “it is preferable to have at least ten judges per study, in order to access the ‘collective expertise’ of judges.”

The results show that SSR values tend to be systematically lower than SHR values, in line with previous observations. One way to correct for this underestimate is using the Spearman–Brown prophecy formula; the results of applying this formula to the SHR values is shown in Fig. 8a. While the rationale for applying the Spearman–Brown correction relies on properties of a test built from items – that does not apply in this case – the results nevertheless show that SSR is more closely aligned with the Spearman–Brown-corrected SHR.

#### SSR as an estimate of inter-group correlation

Next, to determine whether SSR is a suitable proxy for inter-rater reliability, we used the data from our sample to run a simulation. The goal of the simulation was to determine how well SSR estimates the expected correlation of the current scores with scores produced by another equal-sized group of assessors. To do this, for each study we split the assessors into two groups at random (to simulate having two similarly sized groups of assessors), fit the Bradley–Terry model separately with each group’s judgements, and computed the correlation between the scores produced by the two groups, as well as the SSR for one of the groups. After repeating this process 100 times, the median of the correlations gave the SHR measure, and the median of the SSRs gave the “$\text {SSR}_\text {HALF}$” measure. We used 100 repetitions for consistency with our approach to computing SHR; this balanced the cost of repeating the process (which is computationally intensive for the largest datasets) with a desire for stable estimates. We assessed stability across the 100 repetitions using the standard error in the mean; we found that this was typically below .01 (for SHR: median .004, maximum .040; for $\text {SSR}_\text {HALF}$: median .003, maximum .017).

As shown in Fig. 8(b), the $\text {SSR}_\text {HALF}$ was significantly predictive of SHR ($b = 1.40$, $t(62) = 10.41$, $p < .001$) and also explained a significant proportion of variance in SHR ($R^2=.64$, $F(1, 62) = 108.4$, $p < .001$). The resemblance of this relationship with the Spearman–Brown correction in Fig. 8(a) further suggests that the correction formula might reasonably be applied to SHR.

The results from this simulation also showed how likely it was to obtain SHR values above the commonly used .70 threshold, for different values of $\text {SSR}_\text {HALF}$. When $\text {SSR}_\text {HALF}$ was above .8, the SHR measure was above the commonly used .7 threshold in 32 out of 35 cases (91%). For $\text {SSR}_\text {HALF}$ above .85, this rose to 25 out of 26 cases (96%). This suggests that researchers should aim for SSR of .8 or greater, as a proxy for acceptable inter-rater reliability.

## Discussion

We conducted a meta-analysis on comparative judgement (CJ) studies in order to better understand the range of study designs used, and to understand how study design impacts the reliability of outcomes. We obtained the original comparison data from 101 CJ sessions for our analysis.

We found that study characteristics varied widely, in terms of the numbers of representations ($N_R$), assessors ($N_A$) and comparisons ($N_C$) used. However, bar a handful of outliers, the overwhelming majority of judgement sessions had $N_R<1000$, $N_A<1000$, and $N_C<10,000$. We also found that the ratios of these characteristics varied widely, specifically representations per assessor ($N_{RA}$), comparisons per assessor ($N_{CA}$) and comparisons per representation ($N_{CR}$). The ratio of most interest to researchers when designing a CJ study is $N_{CR}$ (the number of comparisons per representation), and we found that while over half of the judgement sessions surpassed the discussed threshold of $N_{CR}\ge 20$ (or $CPR\ge 10$), almost half fell short of the threshold.

Reliability estimates were not always reported in the studies, and in any case reported values may have been inconsistent from study to study. For SSR, some authors may have used an erroneous version of the formula (Bramley & Vitello, 2018), while for SHR there are different practices regarding the number of iterations to use, and whether to take the mean or median. Therefore for each study we re-calculated SSR and SHR using the original comparison data. We found that both SSR and SHR varied widely across the judgement sessions, and that, as expected, SSR was generally higher than SHR. Consistent with this, across the 101 judgement sessions, we found $\text {SSR} > .7$ in 96 cases, but $\text {SHR} > .7$ in only 68 cases.

We also investigated how study characteristics influenced reliability for the 64 judgement sessions that did not use an adaptive pairing algorithm. We found that two characteristics, $N_A$ and $N_C$, and one ratio of characteristics, $N_{CR}$, correlated positively with both reliability estimates (SSR and SHR). However, when these characteristics were entered into a forced regression model only $N_{CR}$ remained a significant predictor. This corroborates Verhavert et al.’s (2019) findings based on studies from education research, and justifies many researchers’ focus on $N_{CR}$ when designing CJ studies. We also explored the extent to which proposed different minimum values of $N_{CR}$ resulted in acceptable reliability. Our results suggest that setting $N_{CR}\ge 20$ tends to produce $\text {SSR}\ge .8$ and $\text {SHR}\ge .7$.

We were also interested in how SSR functions as a measure of inter-rater reliability. To investigate this we again focused on the 64 non-adaptive judgement sessions, and compared SSR to SHR, where the latter can be considered to estimate inter-rater reliability. The two measures were highly correlated, $r=.86$, but as noted above SHR tended to be lower than SSR. Recall that SHR is known to be an underestimate of inter-rater reliability, because it involves splitting the decision data into halves and fitting the Bradley–Terry model for each half separately. To try and account for this, we conducted a simulation study in which SSR was also computed using half rather than all of the comparisons ($\text {SSR}_\text {HALF}$), thus giving a fairer comparison with SHR. The outcome of the simulation was that $\text {SSR}_\text {HALF}>.8$ implies $\text {SHR}>.7$ for over $90\%$ of the 64 data sets included in the simulation. In practice, this means that an SSR value exceeding .8 suggests that a similar number of new judgements would be very likely to produce scores that have a correlation above .7 with the current scores.

### Implications for CJ research

Our analysis involved a greater number and a greater variety of studies than the meta-analysis of Verhavert et al. (2019). Nevertheless, our findings and therefore our recommendations are broadly consistent with, but not identical to, those of Verhavert et al. (2019).

Both measures of reliability, SSR and SHR, were highly correlated among the judgement sessions that used non-adaptive CJ algorithms. Therefore, we suggest that researchers need only report SSR and not SHR. This has the advantage of avoiding the need for the larger numbers of assessors and comparisons required to compute SHR. However, since SSR tends to be higher than SHR, we suggest that a higher threshold than the usual .7 should be adopted. Based on the findings from our meta-analysis, we recommend that researchers consider the threshold for acceptable reliability to be $\text {SSR}\ge .8$, since this is very likely to correspond to inter-rater reliability exceeding the usual .7 threshold.

We also found that of the study characteristics we considered, only the ratio characteristic $N_{CR}$ impacts the reliability of CJ studies. To achieve the recommended $\text {SSR}\ge .8$, our results suggest that researchers use $N_{CR}\ge 20$ ($CPR\ge 10$). In other words, researchers should multiply $N_R$ by 10 or more when deciding how many comparisons to collect.

We note that our recommended values for $N_{CR}$ and SSR do not guarantee that inter-reliability is acceptable. In $9\%$ of the data sets in our meta-analysis that achieved $\text {SSR}\ge .8$, we still observed $\text {SHR}<.7$. Where researchers have the resources and time to collect many judgements, estimating and reporting SHR can help maximise confidence that outcomes are reliable. Thus, we recommend reporting SHR when CJ is being applied in a substantially novel way (for example, using representations that may be difficult for assessors to judge). In such scenarios, we recommend using $N_{CR}\ge 20$ (i.e., multiply $N_R$ by 20 or more) so that the split-halves will each satisfy $N_{CR}\ge 10$.

### Limitations and directions for future research

Our results are limited to the 101 comparative judgement sessions for which we were able to obtain decision data. Moreover, the well-known issue of SSR being inflated by adaptivity (Bramley & Vitello, 2018) limited our analyses for RQ2 and RQ3 to only 64 of the 101 CJ sessions. A larger sample of comparative judgement sessions might have impacted our findings and conclusions. Furthermore, while we have expanded the scope of our meta-analysis compared to Verhavert et al. (2019) to include a range of academic disciplines, our sample of comparative judgement sessions remained dominated by education applications (76%). This may simply reflect the relative popularity of CJ in education research, given that our search was not restricted to any academic discipline and included a range of commonly used terms. Nevertheless, it may be that some research areas using CJ were missed in our search. In any case, as CJ becomes more widely adopted across disciplines, it would be worthwhile for further meta-analyses to re-evaluate our conclusions.

One reason that a balanced spread of disciplines might produce different results is that the range of characteristics $N_R$, $N_C$, $N_A$, as well as their ratios, might be differently distributed compared to the sample of studies included here. For example, in education studies, the representations are commonly student work, and so $N_R$ often reflects typically cohort sizes such as $\approx 30$ (a typical school class) or $\approx 100$ (a typical lecture size or school year). By contrast, other disciplines commonly focus on small $N_R$ (as in marketing, where just a handful of brands might be of interest), or large $N_R$ (as in citizen science projects, where members of the public are recruited to help produce and process enormous data sets). Moreover, there may be a particular issue with studies in which $N_R$ is very large, due to the number of possible pairings increasing proportional to $N_R^2$, whereas our recommendation is to increase $N_C$ directly proportional to $N_R$. Consequently, as $N_R$ increases, $N_C$ is an ever smaller percentage of possible pairings. For future research, it would be worthwhile to have access to data from studies with large $N_R$, to investigate if this issue is observed in practice.

A related limitation of the present study is that we did not consider the impact of all possible study characteristics on CJ outcomes. For example, we are aware of variation in the nature of the assessors, who might be experts, members of the public, paid employees, volunteer participants, or (in the case of education) students engaging in peer assessment. Moreover, there are likely to be characteristics that vary in a discipline-specific way; a likely candidate is the nature of the representations. Our sample included varied types of representations (in addition to the examples of student work that were common in education studies), including food and drink samples (Luckett et al., 2020), images of computer interfaces (Vanderdonckt et al., 2019), and the names of ministries in the Brazilian government (Zucco et al., 2019). It would be worthwhile for future research to expand on our analysis, using a sample of studies with even greater diversity in the types of representations used, and developing a way to classify their features (e.g., their complexity). This would enable exploration of how the representations’ features, and the types of assessors involved, may influence reliability.

A further limitation is that our analysis considered only SSR and SHR as measures of reliability. We focused on these measures due to their widespread use, and the existing interest in the relationship between them (as explained in Section “Estimating reliability”). However, other analyses may be informative. In particular, Wu et al. (2022) proposed a framework of model diagnostics which can be used to check whether various assumptions of the Bradley–Terry model are satisfied (for instance, the “consensus assumption”, that assessors “generally agree on the ranking of popularity of the objects and vote according to their Bradley–-Terry scores”, p. 470). While beyond the scope of the present work, applying this diagnostic framework to the 101 datasets we have collected could contribute further insight into the robustness of CJ methods across a range of applications, and moreover, could help to develop the diagnostic framework.

Finally, our meta-analysis is arguably narrow in a technical sense. First, we only included studies in which assessors are forced to make a choice and ties are not allowed, although disallowing ties seems to represent common practice across disciplines (Issa Mattos & Martins Silva Ramos, 2022). Second, our results for research questions 2 and 3 are based on fitting the Bradley–Terry model. Although this again seems to be the most common practice across disciplines (Hamilton et al., 2023) it is not the only method for modelling decision data and other methods are being proposed, including Bayesian methods (Issa Mattos & Martins Silva Ramos, 2022; Gray et al., 2024) and Elo methods (e.g., Routh et al., 2023). Third, our meta-analysis did not consider situations in which pre-existing data about the representations can be fed into the Bradley–Terry model along with the decision data. For example, this has been done in applications of CJ to modelling sports outcomes to account for home advantage (e.g., Cattelan et al., 2013), and recently Seymour et al. (2022) used spatial parameters in human geography applications to reduce the number of comparisons required. We expect such technical developments to continue, and any subsequent improvements in the accuracy or efficiency of CJ methods would have implications for our findings and recommendations.

### Conclusion

This paper provides researchers using CJ with guidance for evaluating and reporting reliability estimates. Our results broadly vindicate the practice of researchers, and support the overall findings of a previous meta-analysis. In practical terms, our findings should provide researchers with reassurance that reporting SSR is adequate in most cases, provided they use the threshold of .8 rather .7. Moreover our results suggest that $N_{CR}\ge 20$ (i.e., collecting ten times as many decisions as there are items to be judged) should suffice in most cases; this advice is more lenient than Verhavert et al. ’s (2019) suggestion. Nevertheless, CJ methods continue to receive attention and to be developed across disciplines, and as this happens guidelines for evaluating and reporting reliability should continue to be updated.

## Data Availability

Materials and analysis code are available at https://doi.org/10.17605/OSF.IO/M5WTZ.

## Footnotes

1 In particular, to make the Bradley–Terry model identifiable, an additional constraint must be introduced (Wu et al., 2022, Section 2.1). The sirt implementation uses the *sum constraint*, which says that the scores should sum to 0. This approach is widely used, but another option is to choose one of the scores to set to 0 (Turner & Firth, 2012)

2 Two of the characteristics not included in our analysis were: the nature of the assessors (for example, whether the assessors were students’ professors or peers), and format of the representation (for example, static text or audiovisual). We return to these characteristics, and our decision to omit them from our analysis, in the Discussion section. The third characteristic that was not relevant was “feedback”, which related to whether assessors were asked to provide feedback along with each judgement.

3 In one case, an author noted that they had the data but could not share it with us as the paperwork required by their institutional review board was too onerous in the time available.

4 this is equivalent to Equation 1; see Bramley (2015, p. 5)

5 Recall that $N_{CR}$ is computed as double the ratio, i.e., $N_{CR}=2\frac{N_C}{N_R}$, to reflect the fact that each judgement contributes a comparison for each of the representations involved.

6 Note that, for instance, $\log (N_{RA})=\log (N_R)-\log (N_A)$, so adding $\log (N_{RA})$ to the model would not add any new information.

## Code availability

Materials and analysis code are available at https://doi.org/10.17605/OSF.IO/M5WTZ.

## Declarations

### Open practices statement

Materials and analysis code are available at https://doi.org/10.17605/OSF.IO/M5WTZ. None of the reported analyses were preregistered.
